# Supplementary material for: Parkinson’s disease-derived α-synuclein assemblies combined with chronic-type inflammatory cues promote a neurotoxic microglial phenotype
Source: J Neuroinflammation. 2024 Feb 21;21:54. doi: 10.1186/s12974-024-03043-5 (PMC10882738; doi:10.1186/s12974-024-03043-5)
Supplement: Supplementary file 1 — Additional file 1: Figure S1. Morphology of patient-derived αSYN assemblies. Electron micrographs of αSYN assemblies generated after the 3rd cycle of amplification by PMCA from 4 PD (#258, 341, 405, 523) and 4 DLB (#163, 330, 362, 385) patients. Note the relatively flat and twisted appearance of PD-derived assemblies compared to the cylindrical and not-twisted shape of those derived from DLB patients. Scale bar = 200 nm. Figure S2. TPFPD-specific gene ontology term enrichment analysis. Bubble chart showing the enrichment of the GO terms in DEG of TPFPD-treated microglial cells (adjusted p < 0.05). Bubble size indicates the number of genes annotated in the indicated GO term enrichment. Colors represent pathway enrichment (% of overlapping genes) in TPFPD-exposed cells. Figure S3. TPFPD-related chronic-type inflammatory stimulation does not induce microglial cell death. Quantification of LDH release in microglial cells exposed or not (NSC) to FPD (1.5 µM), TNFα + PGE2 + FPD (TPFPD), LPS (10 ng/mL) or 1% Triton X-100 (as a positive control). Any treatment but 1% Triton X100 result in cell membrane disruption-associated LDH release. Bars are means ± SEM (n = 3–6). *p < 0.05 vs. all other conditions (Tukey’s test). [file 12974_2024_3043_MOESM1_ESM.docx]

**Additional File 1.**

**Additional Figures S1 to S3**


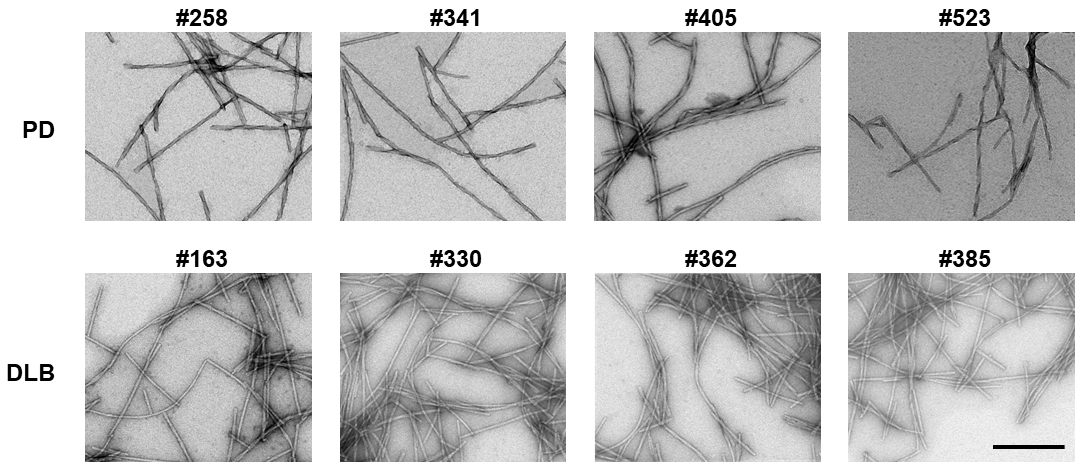


**Figure S1. Morphology of patient-derived αSYN assemblies.** Electron micrographs of αSYN assemblies generated after the 3rd cycle of amplification by PMCA from 4 PD (#258, 341, 405, 523) and 4 DLB (#163, 330, 362, 385) patients. Note the cylindrical and twisted appearance of PD-derived assemblies compared to the flatted and not-twisted shape of those derived from DLB patients. Scale bar = 200 nm.


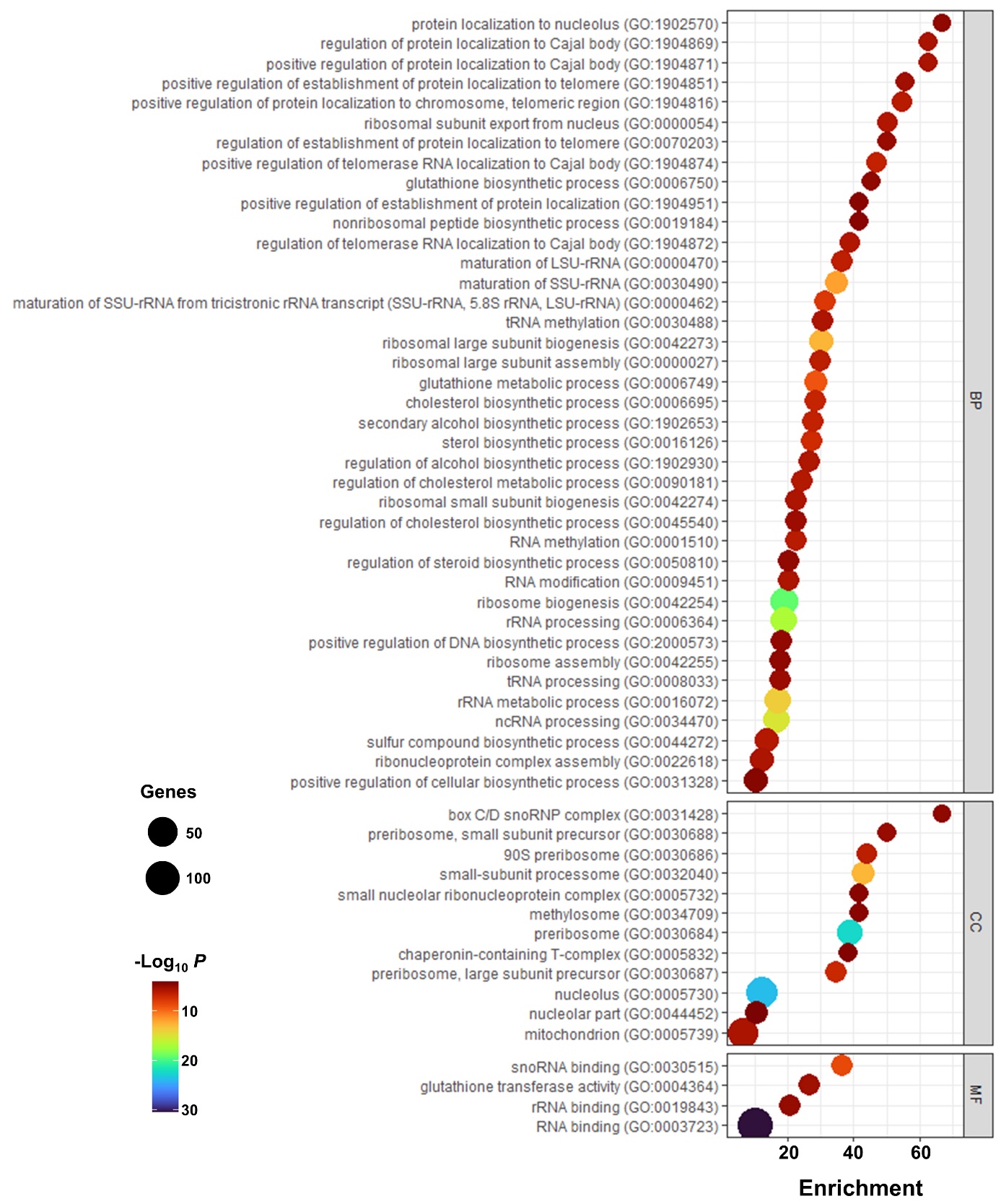


**Figure S2. TPF^PD^-specific gene ontology term enrichment analysis.** Bubble chart showing the enrichment of the GO terms in DEG of TPF^PD^-treated microglial cells (adjusted *p* < 0.05). Bubble size indicates the number of genes annotated in the indicated GO term enrichment. Colors represent pathway enrichment (% of overlapping genes) in TPF^PD^-exposed cells.

**
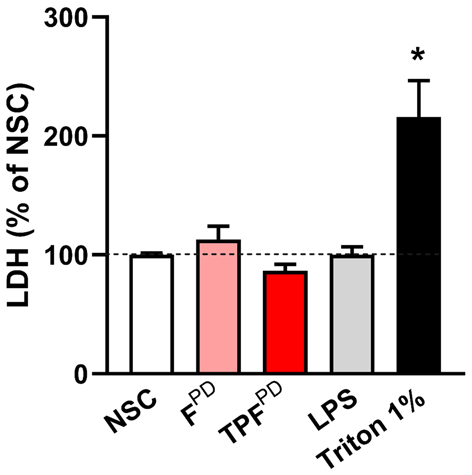
**

**Figure S3. TPF^PD^-related chronic-type inflammatory stimulation does not induce microglial cell death.** Quantification of LDH release in microglial cells exposed or not (NSC) to F^PD^ (1.5 µM), TNFα+PGE_2_+F^PD^ (TPF^PD^), LPS (10 ng/mL) or 1% Triton X-100 (as a positive control). Any treatment but 1% Triton X100 result in cell membrane disruption-associated LDH release. Bars are means ± SEM (n = 3-6). **p* < 0.05 vs. all other conditions (Tukey’s test).
